# Flame Retardancy Properties and Rheological Behavior of PP/DiDOPO Conjugated Flame Retardant Composites

**DOI:** 10.3389/fchem.2022.933716

**Published:** 2022-06-14

**Authors:** Daohai Zhang, Xiaoyu Shang, Jiyong Luo, Junzhuo Sun, Fang Tan, Dongmei Bao, Shuhao Qin

**Affiliations:** ^1^ School of Chemical Engineering of Guizhou Minzu University, Guiyang, China; ^2^ National Engineering Research Center for Compounding and Modification of Polymer Materials, Guiyang, China

**Keywords:** DiDOPO, conjugated structure, polypropylene, flame retardant efficiency, rheological behavior

## Abstract

A bridged 9,10-dihydro-9-oxa-10-phosphaphenanthrene-10-oxide derivative (DiDOPO) with conjugated structure was utilized as a novel conjugated flame retardant, Polypropylene(PP)/DiDOPO conjugated flame retardant composites were papered by being melt-extruding with a twin-screw extruder. The flame retardant efficiency of PP/DiDOPO conjugated flame retardant composites were investigated by cone calorimetry, limiting oxygen index (LOI), vertical burning test (UL-94). Besides, the rheological behavior of PP/DiDOPO conjugated flame retardant composites are measured by ARES rheometer. The results showed that when the content of DiDOPO with conjugated structure was 16 wt%, the LOI values of PP/DiDOPO conjugated flame retardant composites was 24%, and PP/DiDOPO conjugated flame retardant composites reaches V-0 grade. The heat release rate (HRR), total heat release rate (THR) and CO_2_ of PP/DiDOPO conjugated flame retardant composites decreased, so PP/DiDOPO conjugated flame retardant composites had excellent flame retardant effect. Rheological analysis results indicated that DiDOPO with conjugated structure suppressed the melt dripping of PP/DiDOPO conjugated flame retardant composites by enhancing the melt stability. The results showed that the DiDOPO with conjugated structure can significantly enhance the flame retardancy effect of PP/DiDOPO conjugated flame retardant composites. In addition, the materials PP/DiDOPO might be with low conductivity and charge transport mobility.

## Introduction

**FIGURE 1:**
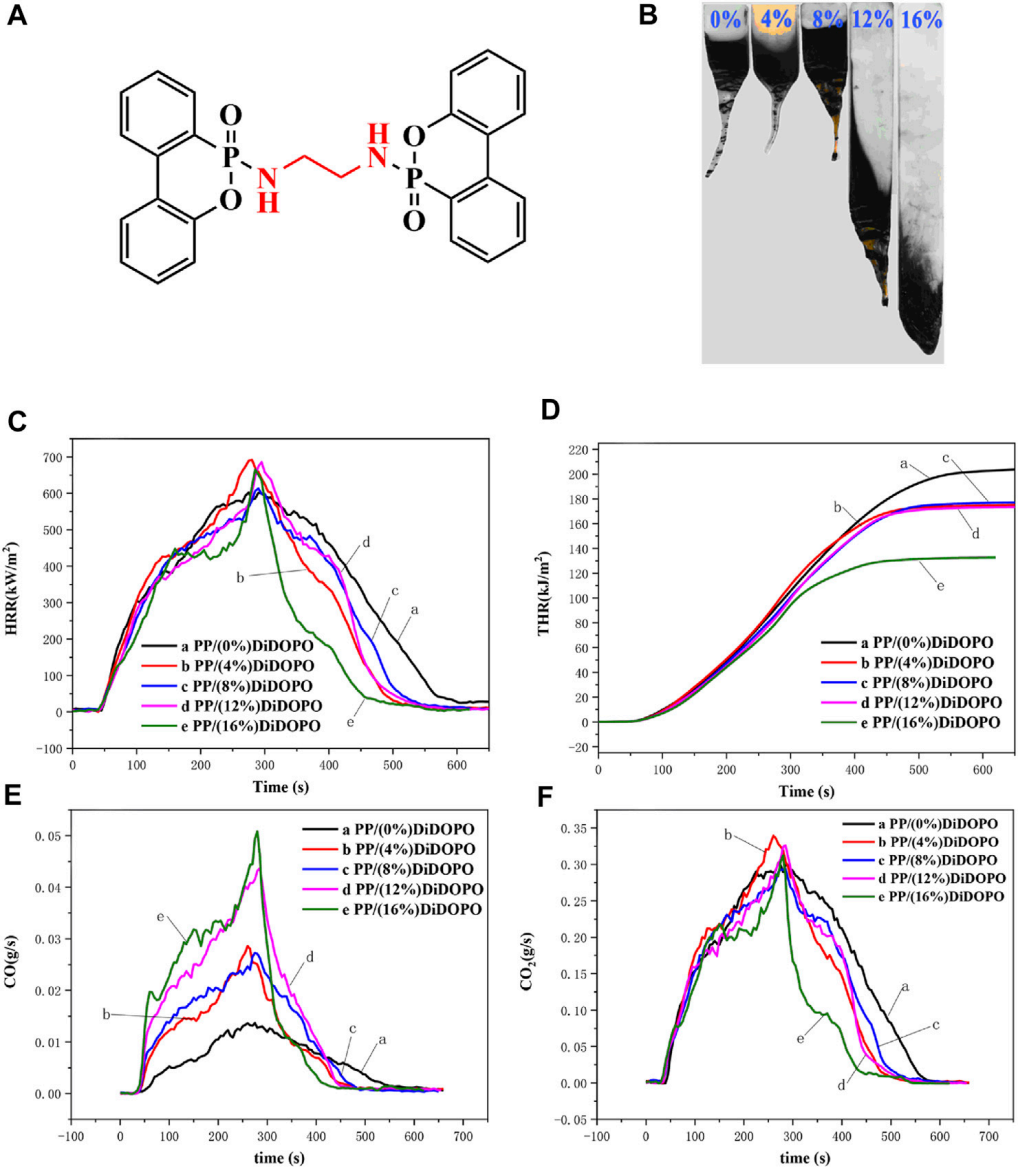
the DiDOPO flame retardant with conjugated structure **(A)**, Photos of samples after UL-94 test **(B)** and HRR **(C)**, THR **(D)**, CO **(E)**, and CO_2_ **(F)** yield curves of pure PP and each component PP/DiDOPO conjugated flame retardant composites.

Polypropylene (PP) have widely been applied in electronic casings, building materials, automotive products, and furniture due to their desirable properties, such as cost-effective, ease processing, low density, and excellent mechanical properties (Wu et al., 2020; Park and Lee, 2021). Unfortunately, because of PP has poor flame retardancy properties, when using in some fields that requires flame resistance is limited. Recently, halogen-based flame retardant has caused great damage to the ecological environment, so the research and application of halogen-free flame retardant in PP composites is extremely urgent. At present, the halogen-free flame retardants are mainly in metal compounds and intumescent flame retardants. Among them, intumescent flame retardants for PP are the more effective. Intumescent flame retardants primarily composed of phosphorous, nitrogen and polyalcohols, but intumescent flame retardants is easy to absorb moisture, the dispersion of the intumescent flame retardants is uneven (Xia et al., 2018; Qi et al., 2020; Wang et al., 2021). Thus, to enhance the flame retardancy properties of PP, we exploring a new flame retardant systems that is DiDOPO with conjugated structure (Figure 1A). The DiDOPO with conjugated structure is a 9,10-dihydro-9-oxa-10-phosphaphenanthrene-10-oxide(DOPO) derivative, DOPO derivatives with conjugated structure have received extensive attention in the past few, which present low conductivity and poor charge transport mobility. In addition, these derivatives also exhibit excellent flame retardancy efficiency and environmental friendliness (Wang et al., 2011; Salmeia and Gaan, 2015). At present, DOPO derivatives with conjugated structure have been widely used in polymer such as polyester, polylactic acid, polyamide, epoxy resin and so on (Liu et al., 2013; Xie et al., 2013; Yang et al., 2015; Wang et al., 2016; Cai et al., 2017), but DOPO derivatives with conjugated structure are used less in PP.

In this study, PP/DiDOPO conjugated flame retardant composites were papered by a twin-screw extruder. The flame retardant properties and rheological behavior of PP/DiDOPO conjugated flame retardant composites were investigated by cone calorimetry, limiting oxygen index (LOI), vertical burning test (UL-94) and ARES rheometer.

## Materials and Methods

### Materials

PP (commercial name: K9026) was supplied by SINOPEC Beijing Yanshan Petrochemical Co., Ltd. (China). The PP pellets were dried in flowing air at 60°C for 12 h before use. The bridged 9,10-dihydro-9-oxa-10-phosphaphenanthrene-10-oxide derivative (DiDOPO with conjugated structure, commercial name: HTP-6123)was purchased by Guizhou Yuanyi Phosphorus Series New Material Co., Ltd. (China). All materials were used without any further purification.

### Preparation of Flame Retardant PP Materials

PP, and dry DiDOPO (4, 8, 12, and 16 wt%) were first dry-mixed (shaken in a bag to combine), respectively, followed by being melt-extruding with a twin-screw extruder (CTE-20, Coperion Machinery Co., Ltd., China). The six heating zones were set to 190°C, 200°C, 205°C, 205°C, 210°C and 205°C, and the screw speed were set at 320 r/min. The purchased compound of DiDOPO were dried in a vacuum oven at 80°C for 12 h to remove any residual moisture, followed by molding on an injection molding machine (SE-130; DongHua Machinery Co., Ltd., China) at 240°C into various specimens for testing and characterization.

### Characterization Methods

The combustion test was conducted on a cone calorimeter test using an FTT cone calorimeter (UK) in accordance with ISO 5660-1 standard. The specimens were prepared with sizes of 100*100*6 mm^3^ and tested under a heat flux of 50 kW/m^2^. Each measurement was performed twice, and the results were averaged.

Limiting oxygen index(LOI) measurements were performed on an oxygen index flammability gauge(HC-2C) according to ASTM D 2863-97 standard with a sample dimension of 100 mm*6.5 mm*3.2 mm^3^.

The vertical burning test (UL-94) was carried out on a SH5300 type instrument (Guangzhou Xinhe Electronic Equipment Co., Ltd., China) was conducted with sample sizes of 130*10*3.2 mm^3^ in accordance with the ASTM UL 94-2006.

The linear viscoelastic behavior of the PP/DiDOPO composites was analyzed by a dynamic oscillatory rheometer in the melt state. A rotation rheometer (ARES-G2, TA Instruments Corp., United States) equipped with 25 mm diameter parallel plates geometry was employed for the rheological tests. Samples were directly loaded and molded between the plates and rheological tests were carried out at 190°C with a gap distance of 0.8∼1 mm. The linear viscoelasticity test has a strain setting of 0.1% and a scanning frequency range of 0.1 rad/s to 500 rad/s.

## Results and Discussion

### The Flame Retardant Properties of PP/DiDOPO Conjugated Flame Retardant Composites

The effects of DiDOPO with conjugated structure on the flame retardancy properties of PP/DiDOPO conjugated flame retardant composites were investigated by vertical burning test (UL-94) and limiting oxygen index test (LOI). The results UL-94 and LOI for PP/DiDOPO conjugated flame retardant composites are shown in Figure 1 and Table 1. As shown in Table 1, when the content of DiDOPO with conjugated structure was 16 wt%, the PP/DiDOPO conjugated flame retardant composites achieved the UL-94 V0 level. It can effectively inhibit the droplet drop phenomenon of PP for the addition of DiDOPO with conjugated structure. As shown in the Figure 1B, PP/DiDOPO conjugated flame retardant composites still have a droplet drop phenomenon, and the droplets of PP/DiDOPO conjugated flame retardant composites can take away heat and had a certain flame retardant effect. But the droplets of PP/DiDOPO conjugated flame retardant composites were weakened. What’s more, the LOI value PP/DiDOPO conjugated flame retardant composites increased from 18.8% to 24.0%. It can be seen that the DiDOPO flame retardant with conjugated structure has high flame retardant efficiency for PP. This might be explained by that the conjugated materials DiDOPO present low conductivity and low charge transport mobility.

**TABLE 1:** UL-94 and LOI test results of PP/DiDOPO conjugated flame retardant composites.

Samples	LOI(%)	UL-94 (3.2 mm)			
		t_1_/t _2_ ^a^(s)	Dripping	Ignition	Rating
PP/DiDOPO -0%	18.8	^b^BC	Yes	Yes	^c^NR
PP/DiDOPO -4%	19.6	—	Yes	Yes	—
PP/DiDOPO -8%	22	—	Yes	Yes	—
PP/DiDOPO -12%	23.2	3.65/0.28	Yes	Yes	V-1
PP/DiDOPO -16%	24	3.23/0.21	Yes	NO	V-0

a Average combustion times after the first and second applications of the flame.

b BC, burns to clamp.

c NR, not rated.

To further analyze the effect of DiDOPO with conjugated structure on the combustion behavior of PP/DiDOPO conjugated flame retardant composites, cone calorimeter test (CCT) of PP/DiDOPO conjugated flame retardant composites were performed. The test results are presented in Figure 1 and Table 2. As shown in Figure 1C, the pure PP burned out after ignition, and a sharp heat release rate (HRR) peak of pure PP appeared. In the case of all other PP/DiDOPO conjugated flame retardant composites, it was observed that a reduction of time to ignition (TTI) happened, the time to the sharp HRR (PHRR) peak of PP/DiDOPO conjugated flame retardant composites was extended when added DiDOPO with conjugated structure into the PP/DiDOPO conjugated flame retardant composites (Schartel and Hull 2007), which was mainly caused by the decomposition of the DiDOPO with conjugated structure flame retardants. Because the thermal stability of DiDOPO with conjugated structure is relative lower than PP, and the addition of DiDOPO with conjugated structure reduces the thermal stability of the PP/DiDOPO conjugated flame retardant composites. The time peak HRR (t_p_) of the PP/DiDOPO conjugated flame retardant composites increased with increasing the DiDOPO with conjugated structure content. This confirms that the DiDOPO with conjugated structure increases the thermal stability of PP/DiDOPO conjugated flame retardant composites. As shown in Figure 1D, the total heat release(THR) value PP/DiDOPO conjugated flame retardant composites were decreased, when the DiDOPO with conjugated structure was added into conjugated flame retardant composites. What’s more, when the amount of DiDOPO with conjugated structure increased, the THR value of PP/DiDOPO conjugated flame retardant composites decreased. The THR value of PP/DiDOPO conjugated flame retardant composites decreased by 14.9%, when the DiDOPO with conjugated structure content was 15 wt%. In addition, the total smoke rate (TSR) value of the PP/DiDOPO conjugated flame retardant composites increased with increasing the DiDOPO with conjugated structure content (Zhao et al., 2013). This might be due to that the DiDOPO composite present low conductivities and charge transport mobility.

**TABLE 2:** CCT data of PP/DiDOPO conjugated flame retardant composites.

Samples	TTI	PHRR (kW/m^2^)	t _p_ (s)	THR (MJ/m^2^)	Av-HRR (KJ/m^2^)	Av-EHC (MJ/Kg)	TSR (m^2^/m^2^)
PP/DiDOPO-0%	37	602.33	275	203.89	333.60	33.68	2272.82
PP/DiDOPO-4%	34	691.97	280	175.18	324.76	28.09	2933.40
PP/DiDOPO-8%	25	680.79	295	182.52	298.14	24.78	3397.75
PP/DiDOPO-12%	24	689.65	325	175.35	270.85	21.99	4119.14
PP/DiDOPO-16%	23	607.63	350	157.14	248.62	20.79	4040.19

The toxic compounds and smoke were the harm in most cases during the course of a fire, in combination with the Figures 1E,F, the CO production rate of PP/DiDOPO conjugated flame retardant composites was improved, However, the CO_2_ production rate of PP/DiDOPO conjugated flame retardant composites decreased. It is due to the phosphorus-containing compounds which the firing DiDOPO with conjugated structure content releases, and these compounds can inhibit flames and thereby increase the number of components that cause incomplete combustion (Wang et al., 2010). The effective heat of combustion (EHC) expresses the contribution of the active constituents of the material to the heat release in the gas phase during combustion (Qian et al., 2015). As the DiDOPO with conjugated structure content increased, av-EHC PP/DiDOPO conjugated flame retardant composites gradually decreased, that indicates that the content of effective combustion components in the gas phase component decreased. Therefore, the decrease of EHC and CO_2_ production rate, and the increase of TSR and CO production for PP/DiDOPO conjugated flame retardant composites suggest that the quenching activity of DiDOPO with conjugated structure for PP/DiDOPO conjugated flame retardant composites mainly involves gas phase flame retardant effect. The gas phase flame retardant mechanism of PP/DiDOPO conjugated flame retardant composites is mainly due to the phosphorus-containing free radicals which released during the decomposition of DiDOPO with conjugated structure, and these phosphorus-containing free radicals can capture free radicals such as H•, O• or HO• in the flame combustion region to suppress the flame (Fornes and Paul 2003; Cannillo et al., 2006; Buczko et al., 2014). Furthermore, PP/DiDOPO conjugated flame retardant composites produce more CO and less CO_2_ than pure PP during combustion. Among the PP/DiDOPO conjugated flame retardant composites, with the increasing DiDOPO with conjugated structure content, the amount of CO generated increased and CO_2_ generated decreased for PP/DiDOPO conjugated flame retardant composites. The addition of DiDOPO with conjugated structure flame retardant suppresses the combustion of PP/DiDOPO conjugated flame retardant composites, resulting in the flame retardant effect of incomplete combustion, thus increasing the release of CO in combustion fumes and gas phase, and also CO_2_ generated goes down.

### The Rheological Behavior of PP/DiDOPO Conjugated Flame Retardant Composites

**FIGURE 2:**
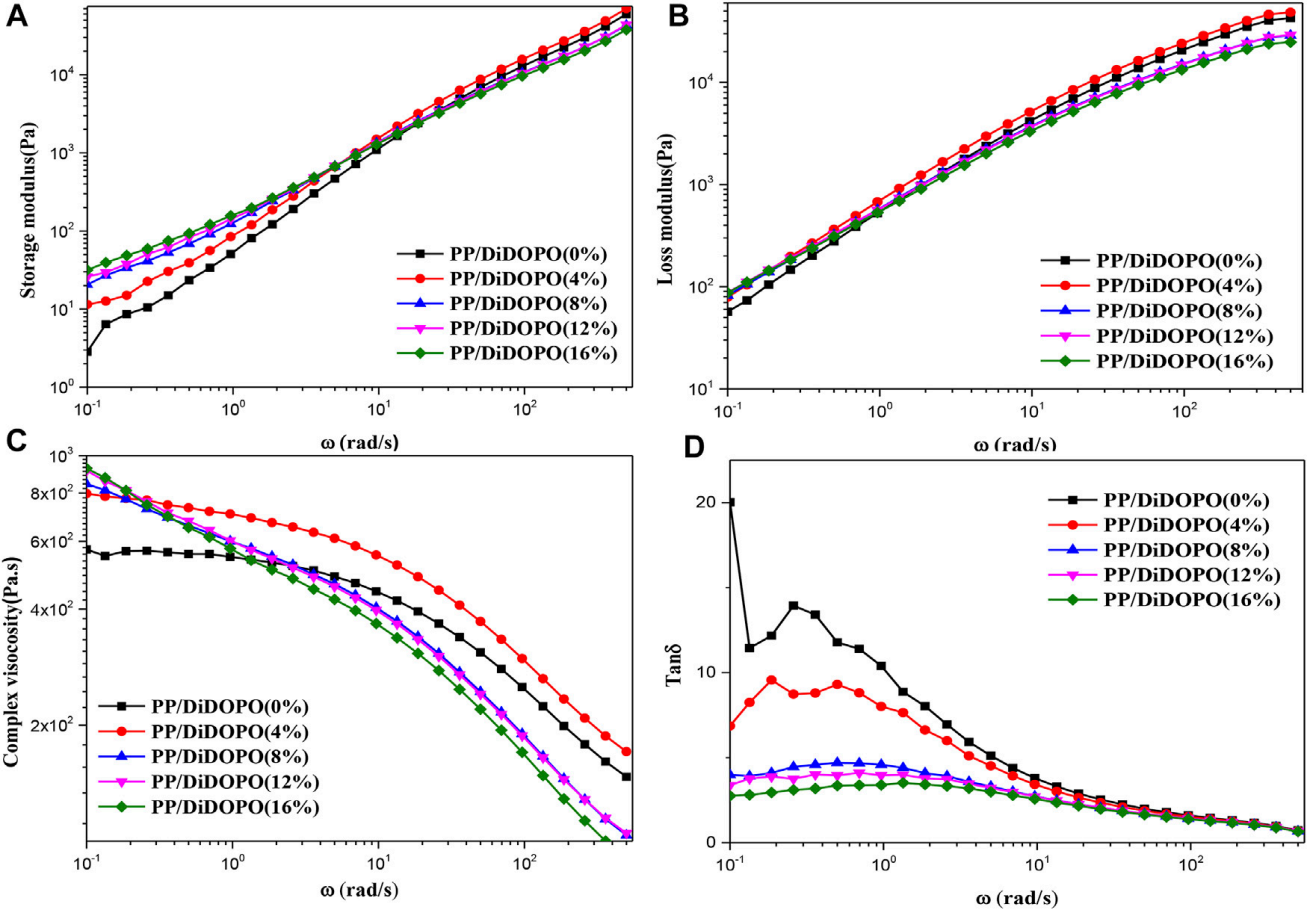
Storage modulus (G′) **(A)**, Loss modulus (G″) **(B)**, Complex viscosity (η*) **(C)** and Tan δ**(D)** for the PP/DiDOPO conjugated flame retardant composites at 190°C.

The storage modulus (G′) of the melt represents the amount of recoverable energy stored in the melt, and loss modulus (G″) represents the magnitude of the non-recoverable energy released by the melt. Melt flow behavior for a material was usually studied by rheology, and the viscoelastic behaviors of the testing samples were investigated by rheology testing to further illustrate the flame mechanism of PP/DiDOPO conjugated flame retardant composites. Figures 2A,B shows the G′and G″of pure PP and PP/DiDOPO conjugated flame retardant composites as a function of the scanning frequency. It can be seen from the Figure 2 that at a higher frequency, the G′ and G″ of PP/DiDOPO conjugated flame retardant composites decreased with the increase of the DiDOPO with conjugated structure content. However, at lower frequencies, the G′ and G″ of PP/DiDOPO conjugated flame retardant composites increased with the increase of the DiDOPO with conjugated structure content.

Complex viscosity η* is a measure of the total impedance of a material to dynamic shear, from storage viscosity (elastic component) and dynamic viscosity (viscous component) consists of two parts, which can be based on the G′ and G″ according to η* = (G′ ^2^ + G″^2^)^1/2^/ω is calculated, where η* is a complex viscosity. The relationship between the complex viscosity (η*) of PP/DiDOPO conjugated flame retardant composites and the frequency is shown in Figure 2C. The results indicated that η* for all the PP/DiDOPO conjugated flame retardant composites gradually decreased during the process. The η* of PP/DiDOPO conjugated flame retardant composites decrease in the entire frequency, which indicated that PP/DiDOPO conjugated flame retardant composites could form a continuous phase to enhance the melt stability, thus it suppress the melt dripping. The tan δ of PP/DiDOPO conjugated flame retardant composites were also significantly affected by the DiDOPO with conjugated structure incorporation, which can be based on the G′ and G″, according to tan δ= G"/G′ is calculated, where tan δ is a loss tangent. From Figure 2D, it can be seen that the tan δ values of the PP/DiDOPO conjugated flame retardant composites gradually shift to a lower with increasing DiDOPO with conjugated structure content. It is important to note that the DiDOPO with conjugated structure promotes the motions of the PP chain segments in the tan δ profile. All the PP/DiDOPO conjugated flame retardant composites had a lower G″ than G′ in the whole frequency region, which indicated a change in rheological behavior, the “solid-liquid” transformation. And that indicated thePP/DiDOPO conjugated flame retardant composites had a liquid behavior under the shear effect.

## Conclusion

In this paper, PP/DiDOPO conjugated flame retardant composites were fabricated by a melt blending method. when the content of DiDOPO with conjugated structure was 16 wt%, the PP/DiDOPO conjugated flame retardant composites achieved the UL-94 V0 level. the LOI value PP/DiDOPO conjugated flame retardant composites increased from 18.8% to 24.0%. The time peak HRR (t_p_) of the PP/DiDOPO conjugated flame retardant composites increased with increasing the DiDOPO with conjugated structure content. the amount of DiDOPO with conjugated structure increased, THR value of PP/DiDOPO conjugated flame retardant composites decreased. THR value of PP/DiDOPO conjugated flame retardant composites decreased by 14.9%, when the DiDOPO with conjugated structure content was 15 wt%. G′ and G″ of PP/DiDOPO conjugated flame retardant composites decreased with the increase of the DiDOPO with conjugated structure content. All the PP/DiDOPO conjugated flame retardant composites had a lower G″ than G′ in the whole frequency region, which indicated a change in rheological behavior, the “solid-liquid” transformation.

## Data Availability

The original contributions presented in the study are included in the article/supplementary material, further inquiries can be directed to the corresponding authors.
